# Ascending colon cancer with synchronous external iliac and inguinal lymph node metastases but without regional lymph node metastasis: a case report and brief literature review

**DOI:** 10.1186/s40792-017-0309-z

**Published:** 2017-02-20

**Authors:** Yuki Kitano, Masafumi Kuramoto, Toshiro Masuda, Daisuke Kuroda, Kenichiro Yamamoto, Satoshi Ikeshima, Ken-ichi Iyama, Shinya Shimada, Hideo Baba

**Affiliations:** 1 Department of Gastroenterological Surgery, Graduate School of Medical Sciences, Kumamoto University, 1-1-1 Honjo, Chuo-ku, Kumamoto, 860-8556 Japan; 2 Department of Surgery, Kumamoto General Hospital, Japan Community Health Care Organization, Kumamoto, 10-10 Tohricho, Yatsushiro, Kumamoto 866-8660 Japan; 3 Department of Surgical Pathology, Kumamoto General Hospital, Japan Community Health Care Organization, Kumamoto, 10-10 Tohricho, Yatsushiro, Kumamoto 866-8660 Japan

**Keywords:** Isolated distant lymph node metastasis, Colon cancer, Synchronous

## Abstract

Lymph node metastasis to the iliac or inguinal region of colon cancer is extremely rare. We experienced a case of ascending colon cancer with synchronous isolated right external iliac and inguinal lymph node metastases but without any regional lymph node metastasis. An 83-year-old woman was admitted to our hospital due to anemia. Colonoscopy and computed tomography revealed an ascending colon cancer and also right external iliac and inguinal lymph node swelling. Further examination by F-deoxyglucose positron emission tomography strongly suggested that these lymph nodes were metastatic. Right hemicolectomy with lymph node dissection along the superior mesenteric artery, and right external iliac and inguinal lymph node dissection were performed. Histological examination revealed that both lymph nodes were metastasized from colon cancer, and there was no evidence of regional lymph node metastasis. The patient has shown no sign of recurrence at 27 months after surgery.

## Background

Distant metastasis of cancer is considered to be a systematic disease, and curative surgical treatment is not generally applied. As for colorectal cancer, however, complete resection of the hepatic or pulmonary metastatic lesions has been demonstrated to improve the survival span, and this approach is regarded as one of the effective ways of potentially curative therapies even for patients with such distant metastasis [1–3]. On the other hand, although there are several reports about the long-term survival of colorectal cancer patients after removal of metastatic lymph nodes, the actual efficacy of the resection of such distant nodes remains unclear [4–7].

In this report, we describe an extremely rare case of ascending colon cancer with synchronous isolated right external iliac and inguinal lymph node metastases with neither regional lymph node metastasis nor distant hematogenous metastasis, for whom a potentially curative operation which included distant metastatic lymph node dissection was performed.

## Case presentation

**Fig. 1:**
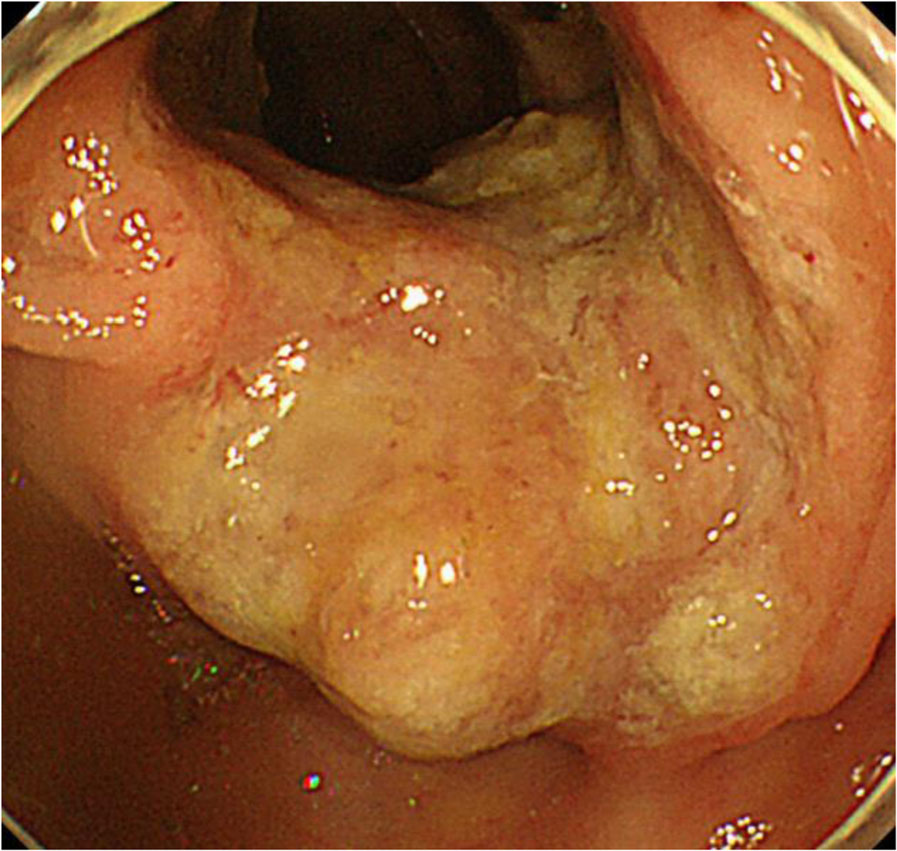
Colonoscopy revealed a type 2 tumor in the ascending colon

**Fig. 2:**
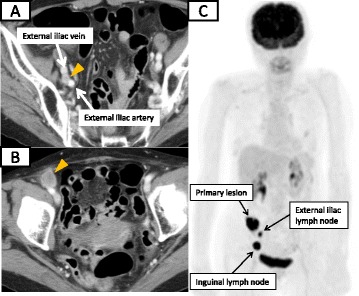
CT shows the enlarged right external iliac (**a**) and inguinal (**b**) lymph nodes, and FDG-PET shows a significant accumulation of FDG into the primary colon lesion with the SUV of 17.9 and external iliac and inguinal lymph nodes with SUV of 9.1 and 25.1, respectively (**c**). The *yellow arrowheads* indicate lymph node

An 83-year-old woman visited our hospital complaining of dizziness. A hematobiochemical examination revealed that the serum level of the hemoglobin was 7.0 g/dL. There were no other abnormal data, including that for the tumor markers (CEA and CA19-9). A series of further examinations was performed. Colonoscopy revealed an ascending colon tumor (Fig. 1), which was histologically proven to be a colon cancer (well-differentiated adenocarcinoma). Computed tomography (CT) demonstrated not only the ascending colon cancer but also the enlarged right external iliac and inguinal lymph nodes, whereas neither the apparent metastasis to the principle organs nor regional lymph nodes were detected. F-deoxyglucose positron emission tomography (FDG-PET) demonstrated a significant accumulation of 18F-fluorodeoxy glucose in the main colon tumor with the standardized uptake value (SUV) of 17.9 and external iliac and inguinal lymph nodes with SUV of 9.1 and 25.1, respectively, which suggested these were metastatic lymph nodes of an unknown origin (Fig. 2). However, a detailed and careful visual examination of the legs detected no evidence of abnormal finding.

**Fig. 3:**
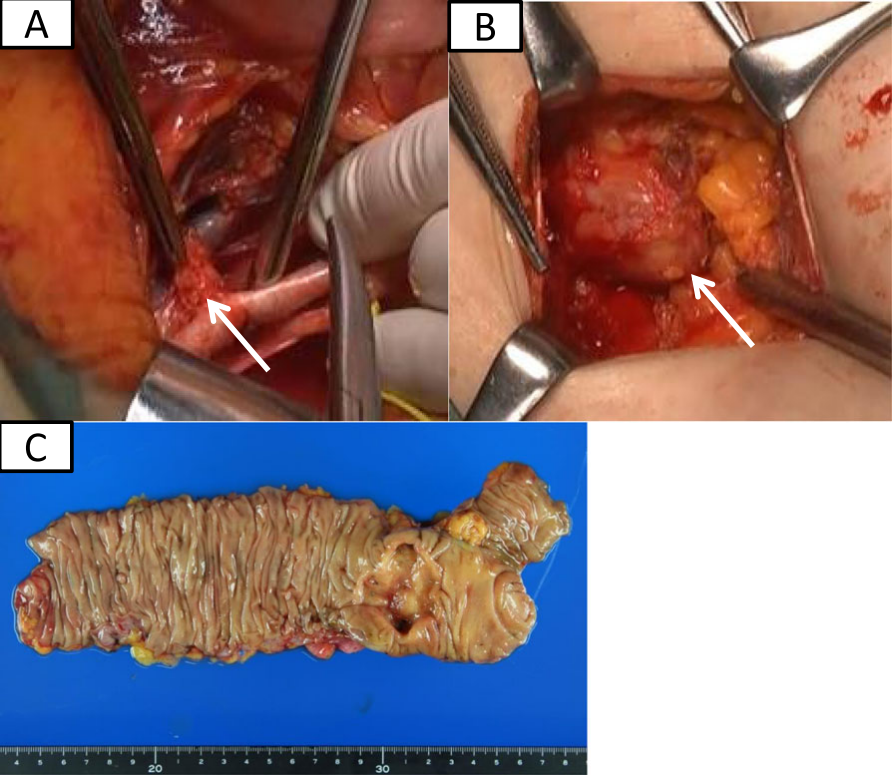
The patient underwent right external iliac (**a**) and inguinal (**b**) lymph node dissection. A surgical specimen showed type 2 tumor (50 × 35 mm) in the ascending colon (**c**)

**Fig. 4:**
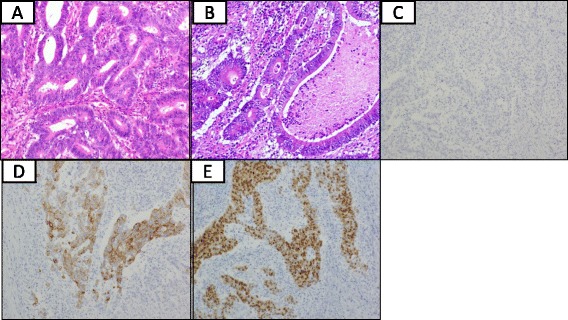
Histopathological examination demonstrated the tumor to be a well-differentiated adenocarcinoma (**a**). The enlarged external iliac and inguinal lymph nodes proved to be metastases of well-differentiated adenocarcinoma (H&E staining) (**b**), and by immunohistochemical examination, cytokeratin 7 was negative (**c**) and cytokeratin 20 (**d**) and CDX2 (**e**) were positive (these figures showed each staining of external iliac lymph node)

A right hemicolectomy with lymph node dissection along the superior mesenteric artery, and right external iliac and inguinal lymph node dissection were performed (Fig. 3a, b), and we confirmed the metastasis of adenocarcinoma in these distant lymph nodes through the intraoperative histological examination. A surgical specimen showed type 2 tumor (50 × 35 mm) in the ascending colon (Fig. 3c) and the histopathological examination demonstrated the tumor to be a subserosa-invasive well-differentiated adenocarcinoma (Fig. 4). As for the external iliac and inguinal lymph nodes, both of them were well-differentiated adenocarcinoma and revealed to be metastasis from the colon cancer by immunohistochemical examination, which revealed a profile that cytokeratin 7 was negative and cytokeratin 20 and caudal-related homeobox 2 (CDX2) were positive (Fig. 4) [8]. Surprisingly, there was no metastasis to the regional lymph nodes and extramural tumor deposits. Finally, the tumor was diagnosed stage IV (T3 N0 M1) colon cancer according to the seventh edition of the International Union Against Cancer TNM classification. At the patient’s request, no adjuvant chemotherapy has been applied. The woman has had no recurrence at 27 months since the operation.

## Discussion

Synchronous metastasis to the right external iliac or inguinal lymph nodes is rare in colon cancer. Furthermore, not only the number of reports on cases of colon cancer with such distant lymph node metastasis is small but also, as far as we know, there are hardly any reports of lymph node metastasis without regional metastasis. To date, only four cases of surgical resection of colon cancer with external iliac or inguinal lymph node metastasis have been reported (Table 1). However, to the best our knowledge, ours is the first case of colon cancer patient with such metastasis without regional metastasis.

**Table 1:** Review of reported five cases (including this case) of colon cancer with external iliac or inguinal lymph node metastasis

Author, year of publication	Age, sex	Primary tumor site	Site of lymph node metastasis	Synchronous or metachronous	pStage (TMN classification)	Outcome and time after surgery
Uehara M, et al., 2007	67, male	Cecum	Right external iliac lymph node	Metachronous	Stage IIIC	Alive (no recurrence) 18 months
Hakeem A, et al., 2009	60, female	Cecum	Left inguinal lymph node	Synchronous	Stage IV (pT3 N2 M1)	Not described
Pisanu A, et al., 2011	62, male	Sigmoid colon	Left inguinal lymph node	Metachronous	Stage IIIB (pT4 N1 M0)	Alive (no recurrence) 8 months
Hara M, et al., 2013	67, male	Cecum	Right inguinal lymph node	Metachronous	Stage IIIB (pT4 N1 M0)	Alive (no recurrence) 36 months
Kitano Y, et al., 2016 (current case)	83, female	Ascending colon	Right external iliac and inguinal lymph node	Synchronous	Stage IV (pT3 N0 M1)	Alive (no recurrence) 27 months

In the current case, the FDG-PET imaging which suspected right inguinal and iliac lymph node metastases led to focusing on finding malignancies in the lower legs. However, despite the detailed observations performed, no evidence of suspected lesions could be detected. Right hemicolectomy with regional lymph node dissection and the right iliac and inguinal lymph node dissection eventually revealed that the distant metastatic lymph nodes had originated from the colon cancer. And what was the most surprising, there was no metastasis to the regional lymph nodes.

In rectal cancer, lymph node metastasis to the iliac or inguinal area is not so rare [9, 10]. Moreover, some reports revealed that there were a few rectal cancer patients having inguinal lymph node metastasis without regional metastasis [11]. The mechanism whereby such metastasis occurs has been suggested as follows: Most of the lymphatic vessels usually run along the arteries and their lymphatic flow usually advances to the root of inferior mesenteric arteries. However, when cancer cells invade to lymphatic vessels, they could cause the proximal lymphatic obstruction and spread with the retrograde lymphatic flow to iliac or inguinal areas [12–14]. However, this supposition might not apply to patients with cancer of the right colon. It is generally accepted that the lymphatic pathways from cecum and ascending colon run toward the root of the superior mesenteric artery. Therefore, based on this viewpoint of the lymphatic flow, lymphatic metastasis to the iliac and inguinal areas without regional metastasis from cecum or ascending colon cancer would seem unlikely to occur [15].

Although there were a few reports on external iliac or inguinal lymph node metastasis of colon cancer, these cases had regional lymph node metastasis [16–19]. Uehara et al. proposed one hypothesis for this rare pattern of lymphatic metastasis. The cancer in the right colon invaded some part of the abdominal wall and then metastasized to the lymph nodes in the region of the right external iliac artery through a lymphatic pathway along the right inferior epigastric artery [16].

In our case, the main tumor did not extend to the abdominal wall and also no apparent microscopic tumor invasion to the serosa could be confirmed. There might have been latent invasion to the abdominal wall, or some unusual lymphatic pathway from the right colon to the right extra-iliac area may have brought about such an improbable lymph node metastasis. However, the actual mechanism of this metastatic pattern still remains to be fully determined.

Although there are several reports on surgical resection for distant lymph node metastasis, the validity of performing node dissection for such cases is controversial. Some studies have suggested the survival benefits of lymph node dissection for the colorectal cancer patients with isolated para-aortic lymph node metastasis, whereas others reported no significant observations in the effectiveness of extensive lymph node dissection [4–6]. We successfully performed a potentially complete resection of ascending colon cancer including dissection of solitary distant lymph node metastasis. The patient is still alive and had no recurrence during the 27 months since the operation. However, a further careful follow-up will be needed to ascertain the validity of this treatment.

## Conclusions

We experienced an extremely rare case of ascending colon cancer with synchronous isolated lymph node metastases in the right external iliac and inguinal region but without regional lymph node metastasis. More observations on a larger accumulation of such patients are required to clarify the proper and effective treatment for such cases, including the resection of solitary distant lymph node metastasis.

## Abbreviations

CA19-9: Carbohydrate antigen 19-9
CDX2: Caudal-related homeobox 2
CEA: Carcinoembryonic antigen
CT: Computed tomography
FDG-PET: F-deoxyglucose positron emission tomography
SUV: Standardized uptake value
